# Left ventricular assist device for end-stage heart failure: results of the first LVAD destination program in the Netherlands: Towards LVAD destination therapy in the Netherlands?

**DOI:** 10.1007/s12471-014-0609-x

**Published:** 2014-10-15

**Authors:** O. C. Manintveld

**Affiliations:** Thoraxcenter, Department of Cardiology, Erasmus Medical Center, PO Box 2040, 3000 CA Rotterdam, the Netherlands

Today, heart transplantation is the golden standard for the treatment of patients with end-stage heart failure. A median survival time of more than 11 years (and 13 years for those surviving the first year) after transplantation will be hard to equal let alone to beat [1]. As a result of donor organ shortage, transplantation can, unfortunately, only be offered to a few patients. Supported by considerable improvements in left ventricular assist devices (LVADs) cardiologists and surgeons in the USA, as well as elsewhere in Europe, are eagerly accepting the LVAD as a long-term therapy (destination therapy).

Historically, LVADs were used as a bridge to a transplantation (BTT) or bridge to recovery in the very few patients whose heart function improved after a period of mechanical support. In less than two decades LVADs have evolved from short-term axial extracorporeal pulsatile devices to smaller intracorporeal continuous flow devices for long-term support. In doing so, the complication rates have dropped considerably and an additional treatment for end-stage heart failure has become a reality, paving the way for LVAD as long-term therapy. Patients who are deemed unfit for transplantation can now be offered an alternative therapy. It is important, however, to realise that the indications of BTT and destination therapy will no longer be mutually exclusive; they may be a moving target. For example, patients on destination therapy may become BTT patients over time due to the fact that a contraindication for transplantation has disappeared: e.g. they have survived their malignancy long enough or their pulmonary pressures have normalised. On the other hand, patients on BTT therapy might become destination therapy patients if they develop a malignancy during the waiting time.

The two leading LVAD devices currently on the market are the Heart Mate II and HeartWare. In the United States the Heart Mate II has been approved for BTT since 2008 and as destination therapy since 2010, while HeartWare is approved as BTT since 2010 and is still awaiting approval for destination therapy. During 2012 more than 40 % of all implants in the USA were designated as destination therapy [2]. In Europe, the current Heart Mate II ratio BTT/ destination therapy is 75 %/25 % (information from Thoratec Corp).

In the Netherlands, mechanical support by an LVAD is only reimbursed by the Health Insurance Companies when used as a BTT. In a recent consensus document, the Netherlands Society of Cardiology and the Netherlands Society of Thoracic Surgery express the opinion that LVAD destination therapy should be part of our therapeutic armamentarium (August 2014). For this purpose they formulated indications and contraindications for destination therapy as well as specific requirements for the implanting teams.

In this issue of the Netherlands Heart Journal, Haeck et al. from Leiden University Medical Centre report their experience in the use of LVADs as destination therapy [3]. They have to be congratulated on being the front runners on this subject in the Netherlands. Without experience in the application of LVADs as BTT they selected 16 patients with end-stage heart failure who remained symptomatic (NYHA class III or more), were on optimal medical therapy and were considered ineligible for transplantation. In these patients, who are at high risk of dying in the short term, they show improvements in NYHA class, in quality of life as well as in 6-min walking distance.

In the Discussion section, Haeck et al. argue that treatment with LVADs results in nearly the same outcome as with heart transplantation (1- and 2-year survival of 80 % and 70 % respectively) [2]. It is suggested that their 6-month survival is in line with the expectations. These statements, however, need nuance. Data about long-term destination therapy with an LVAD are totally absent; therefore a real comparison with heart transplantation cannot be made. In addition, Haeck et al. do not present data about the length of follow-up. In view of the start of their program in November 2010 (Leids Dagblad, 16 November 2010) one could expect that the 1- and 2-year survival rates could have been presented. Of course, such data would have to be interpreted with great caution for two reasons. The number of patients in their study was very small and the team had no experience in the application of LVADs as BTT. The latter must be kept in mind when comparing their results with the published USA data of centres with experience of the implantation of LVADs as BTT. The sixth Interagency Registry for Mechanically Assisted Circulatory Support (INTERMACS) annual report shows that the survival rates of more than 10,000 patients are 80 %, 70 %, 59 % and 47 % after 1, 2, 3 and 4 years, respectively.[2] The 6-month survival of Haeck et al. is already less than the 1-year survival in the INTERMACS registry. Apart from the lack of experience there are various reasons why this may be so. First of all, they included two patients in INTERMACS level 1, who are at the highest risk of dying. Furthermore, they also included a high percentage of patients (81 %) who had to undergo concomitant tricuspid valve annuloplasty. It has been shown that performing a tricuspid valve procedure for severe tricuspid valve regurgitation does not reduce early death or right-sided VAD requirement and that this is associated with worse early postoperative outcomes [4]. Finally, it is known that patients on destination therapy are at higher risk of death than patients on BTT [2].

The rates of adverse clinical events in the studied group (44 %) are somewhat lower than those in the INTERMACS registry (60 % at 6 months) [2]. But the numbers are low and the presented data do not include the in-hospital events. In general, with such a high percentage of adverse clinical events it is not a question of whether patients will have a complication, but rather when. The hope is that in the future, with the use of newer-generation LVADs, these event rates will go down. This is of special importance for patients on destination therapy since they have limited bail out options when problems occur.

Looking into the future, I think that we have to rely on international data to decide upon the approval of LVAD destination therapy as reimbursed care. Results from centres abroad combined with the experience in BTT of the Dutch heart transplant centres make a randomised trial (medical therapy versus destination therapy) unacceptable. LVAD as destination therapy is around the corner but improvement in patient selection, miniaturising of the LVAD, complete internalisation of the power source and placement with minimally invasive surgical techniques are necessary to make this a successful transition. Furthermore, the price per LVAD needs to come down as more are being implanted. As colleagues in Utrecht have recently shown, LVAD destination therapy is still a relatively expensive intervention (€107,600 per quality-adjusted life-years) [5]. Thus costing more than the €80,000 which was suggested as an upper limit by the Netherlands Council for Health and Care in 2006 [6].

An exciting new time is ahead, and we need to keep in mind that as the number of LVAD patients grows we will reach a point that also not-implanting, referring hospitals will have to take over part of the care of LVAD patients as well. A real challenge for all Dutch cardiologists.
